# Circulation of Influenza and Other Respiratory Viruses in Tunisia, 2022–2023 Season

**DOI:** 10.1111/irv.70199

**Published:** 2026-05-11

**Authors:** Bouslah Zoubeir, Bouguerra Hind, Dhaouadi Sonia, Abid Salma, Whitehouse Erin Rachel, El Ghord Hakim, Yazidi Rihab, Ben Mrad Nacef, Miraoui Amal, Bougharriou Ichrak, Ennouri Emna, Ben Slimen Najla, Guebsi Nour El Houda, Toumi Radhouane, Bouabid Leila, Ben Hamida Chokri, Merhabane Takoua, Boussarsar Mohamed, Ben Jemaa Mounir, Menif Khaled, Ben Khelil Jalila, Ben Alaya Nissaf, Ben Salah Afif, Laouini Dhafer, Zureick Kinda, Boutiba‐. Ben Boubaker Ilhem

**Affiliations:** ^1^ Laboratory of Microbiology, National Influenza Centre, National Reference Lab of Respiratory Viruses Charles Nicolle Hospital Tunis Tunisia; ^2^ Faculty of Medicine of Tunis LR99ES09 University Tunis El Manar Tunis Tunisia; ^3^ Studies and Planning Directorate Ministry of Health Tunis Tunisia; ^4^ Faculty of Medicine of Tunis LR01ES04 University Tunis El Manar Tunis Tunisia; ^5^ National Observatory of New and Emerging Diseases Tunis Tunisia; ^6^ US Centers for Disease Control and Prevention Atlanta Georgia USA; ^7^ Primary Health Care Directorate Ministry of Heath Tunis Tunisia; ^8^ Laboratory of Transmission, Control and Immunobiology of Infections, Institut Pasteur de Tunis, University Tunis‐El Manar Tunis Tunisia; ^9^ Intensive Care Department Abderrahmane Mami Hospital Ariana Tunisia; ^10^ Faculty of Medicine of Tunis University Tunis El Manar Tunis Tunisia; ^11^ Research Unit for Respiratory Failure and Mechanical Ventilation UR22SP01 Abderrahmen Mami Hospital Ariana Tunisia; ^12^ Pediatric Intensive Care Unit Béchir Hamza Children's Hospital Tunis Tunisia; ^13^ Department of Infectious Diseases Hedi Chaker University Hospital Sfax Tunisia; ^14^ Faculty of Medicine of Sfax University of Sfax Sfax Tunisia; ^15^ Medical Intensive Care Unit, Research Laboratory No. LR12SP09, Heart Failure Farhat Hached University Hospital Sousse Tunisia; ^16^ Faculty of Medicine of Sousse University of Sousse Sousse Tunisia; ^17^ Intensive Care Unit Regional Hospital of Zaghouan Tunis Tunisia; ^18^ Service de réanimation médicale du CHU Habib Bourguiba de Sfax Sfax Tunisia; ^19^ Department of Family and Community Medicine Arabian Gulf University Zaghouan Tunisia; ^20^ Institut Pasteur de Tunis University Tunis Al Manar Tunis Tunisia

**Keywords:** acute respiratory infections, influenza, respiratory viruses, RSV, SARS‐CoV‐2, sentinel surveillance

## Abstract

**Introduction:**

Influenza sentinel surveillance has been ongoing in Tunisia since 1999. We describe the epidemiology of respiratory viruses during 2022–2023, the first season to include testing for other respiratory viruses, such as respiratory syncytial virus (RSV).

**Methods:**

We analyzed weekly surveillance data from severe acute respiratory infection (SARI) inpatients and influenza‐like illness outpatients from 11 hospitals and 85 clinics in Tunisia. Nasopharyngeal specimens and demographic, clinical, and vaccination data were collected. Specimens were tested by rRT‐PCR for influenza, SARS‐CoV‐2, RSV, and 18 other respiratory viruses. Descriptive statistics were used to summarize case characteristics; group differences were assessed using chi‐squared or Fisher's exact tests.

**Results:**

2038 specimens were collected from unique patients; 1231 (60.4%) were positive for ≥ 1 respiratory virus and 200 (16.2%) were positive for ≥ 2 viruses. Influenza was the most detected (*n* = 445; 21.8%), followed by rhinovirus (*n* = 301, 14.8%), RSV (*n* = 255, 12.5%), and SARS‐CoV‐2 (*n* = 125, 6.1%). Among SARI cases, infections with influenza and SARS‐CoV‐2 were more common in adults ≥ 50 years (61.8% and 71.4%, respectively), while children < 2 years had higher RSV prevalence (83.0%, adjusted *p*‐value = 0.004). 4.9% of patients received a recent influenza vaccine.

**Conclusion:**

The burden of respiratory viruses varied by age, with RSV being more prevalent among younger children and SARS‐CoV‐2 and influenza being more prevalent among older adults. Ongoing sentinel surveillance is essential to monitor priority respiratory pathogens, particularly those with available public health interventions, such as vaccination, to enable timely action and reduce disease burden.

## 1 Introduction

Acute respiratory infections (ARI) constitute a major public health challenge, particularly in low‐ and middle‐income countries (LMICs), due to high incidence rates, mortality, and economic losses [1]. Some studies estimate that viruses cause 50% to 80% of acute respiratory infections, mainly influenza A and B viruses, respiratory syncytial virus (RSV), human rhinoviruses, enteroviruses, and since the COVID‐19 pandemic, SARS‐CoV‐2 [2, 3].

In Tunisia, influenza viruses accounted for nearly 28% of outpatient ARIs in 2012–2015, but a comprehensive national summary of ARIs has not been published since then [4]. Sentinel surveillance for influenza has been conducted in Tunisia since 1999, providing valuable clinical, epidemiological, and virological data over time that contribute to national decision making and global situational awareness [5]. Annual influenza epidemics are particularly devastating and lead to significant morbidity, mortality, and socio‐economic impact due to hospitalization, medication costs, and work absence [6]. Influenza viruses, along with other respiratory viruses excluding SARS‐CoV‐2, circulated at lower‐than‐expected levels during the COVID‐19 pandemic but resurged in 2022 [7, 8, 9]. This shift may be attributed to non‐pharmaceutical interventions during the pandemic, with their relaxation possibly explaining the resurgence 2 years later [7, 8, 9].

Non‐influenza causes of ARIs in Tunisia, which comprise more than two‐thirds of ARIs captured in surveillance, have been understudied, in part because treatment and prevention interventions were not available. Globally, waves of SARS‐CoV‐2 infections continue to cause significant morbidity and mortality, particularly in older adults and those with comorbid conditions [10]. In LMICs, like Tunisia, RSV has had a particularly severe impact on children under 6 months of age [11]. With the novel prevention and treatment interventions for SARS‐CoV‐2 and RSV (e.g., antiviral treatments and vaccinations), extension of surveillance to test for these respiratory viruses remains essential for evidence‐based decision‐making, particularly since the onset of the COVID‐19 pandemic.

In 2022, the Tunisia Ministry of Health leveraged the existing influenza sentinel surveillance system to detect other respiratory pathogens among patients with ARI, in alignment with the World Health Organization's (WHO) integrated surveillance strategy for respiratory pathogens [12]. The main aim of our study was to describe the circulating respiratory pathogens identified through sentinel surveillance during the 2022–2023 season. The secondary aims were to compare epidemiological and virological characteristics between ILI and SARI cases and between three common viruses (influenza, SARS‐CoV‐2, and RSV) among ILI and SARI cases.

## 2 Methods

### 2.1 Surveillance Sites and Case Enrollment

**FIGURE 1:**
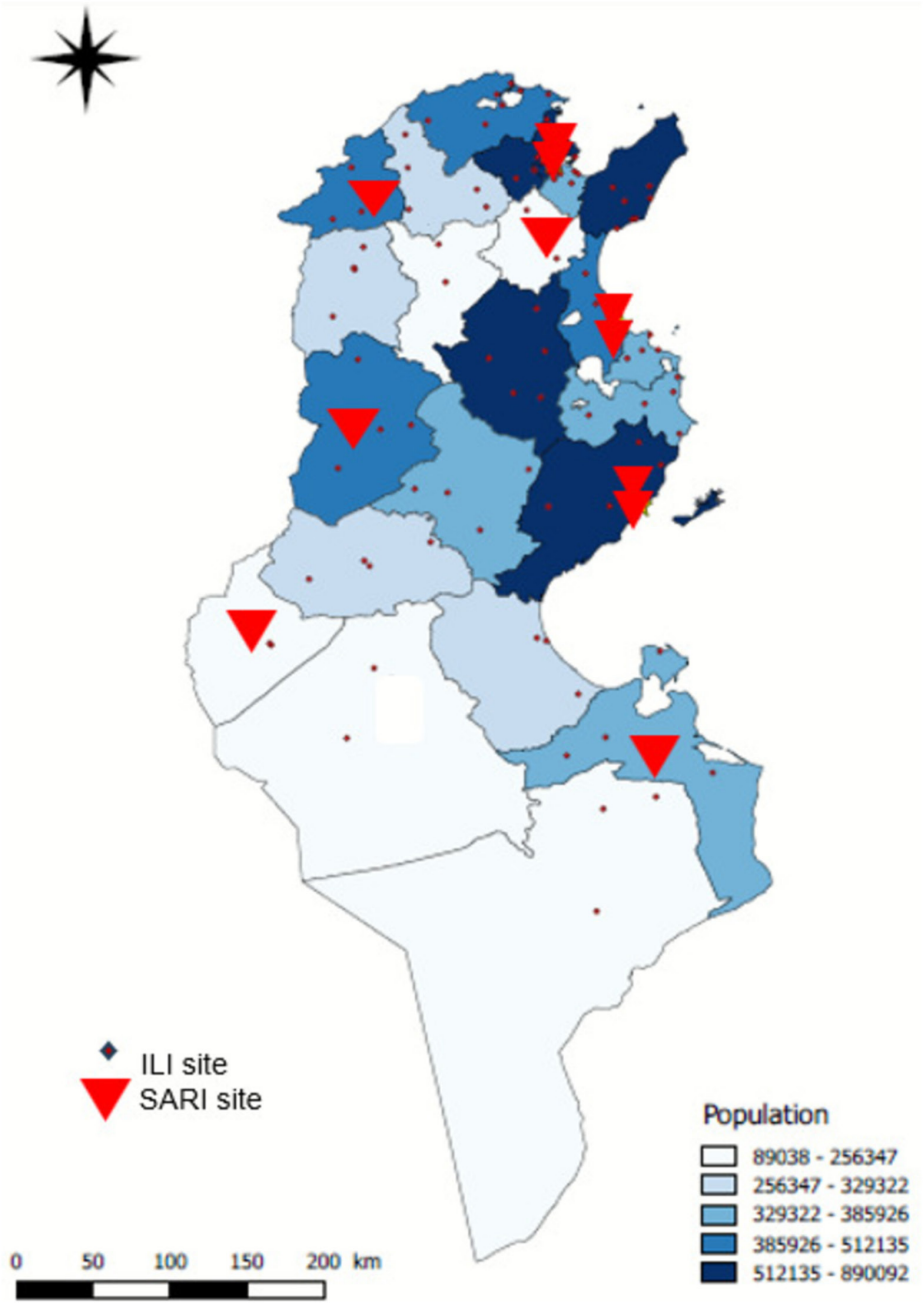
Geographical distribution of influenza‐like‐illness (ILI) and severe acute respiratory infections (SARI)sentinel sites in Tunisia, 2022–2023 season. *Source:* Primary Health care Directorate.

We analyzed national sentinel surveillance data collected in Tunisia from 1st October 2022 to 8th May 2023, following the national guidelines for influenza sentinel surveillance [5]. Sentinel sites included 11 public regional or university hospitals (SARI sites) and 85 public primary healthcare centers (ILI sites) (Figure 1). Within the 11 general (non‐specialty) hospital sites, surveillance was conducted in the intensive care department (*n* = 7, 6 adults and 1 pediatric), pulmonology department (*n* = 3), and infectious disease department (*n* = 1).

A SARI case was defined as per the WHO case definition being a patient who presented with a history of fever or measured fever ≥ 38°C and cough, with onset within the last 10 days and required hospitalization [13]. An ILI case was defined as a patient who presented to an outpatient facility with a measured fever ≥ 38°C and cough, with onset within the last 10 days [13]. All age groups were eligible to be identified as cases.

### 2.2 Specimen and Data Collection

Nasopharyngeal swabs from cases were collected weekly by trained healthcare workers [5]. Specifically, all SARI cases and a subset of ILI cases (generally, 1–10 ILI cases were swabbed per week, per site) were eligible to be swabbed and tested [5]. Swabs were preserved in viral transport medium and refrigerated at 4°C at the sentinel site. Specimens were then transported to the Tunisia National Influenza Center (NIC) in insulated cooler boxes with ice packs, then immediately transferred to a refrigerator and stored at 4°C for a maximum of 72 h before testing at the NIC.

Epidemiologic data on ILI and SARI cases were collected in the national surveillance system's Electronic Information Management System (IMS) and linked with virologic test results. Data collected included age, sex, date of specimen collection, comorbidities (cardiovascular diseases, asthma and other respiratory diseases, and diabetes), current influenza vaccination status, and influenza antiviral treatment received in the last 14 days.

### 2.3 Laboratory Testing

The NIC tested sentinel specimens by real‐time reverse transcriptase polymerase chain reaction (rRT‐PCR) to detect influenza A and B, SARS‐CoV‐2, and other respiratory viruses.

The United States Centers for Disease Control and Prevention's (US CDC) Influenza SARS‐CoV‐2 Multiplex Assay (Flu SC2) was used to detect influenza A, influenza B, and SARS‐CoV‐2 [14]. For influenza‐positive specimens, a US CDC subtyping assay for Influenza A viruses (H1N1 pdm09 and H3N2 subtypes) and a lineage assay for influenza B viruses (Yamagata and Victoria lineages) was used [15].

Additionally, all specimens were also tested using the FTD (Fast Track Diagnostics) Respiratory Pathogens 21 assay according to the manufacturer's instructions (Siemens Healthcare Diagnostics Inc.). This multiplex assay allows the simultaneous and qualitative detection of influenza A virus, influenza A virus H1N1pdm09 swine‐lineage, influenza B virus, human rhinovirus, human coronaviruses 229E, NL63, HKU1, and OC43, human parainfluenza viruses 1 to 4, human metapneumoviruses A and B, human bocavirus, *Mycoplasma pneumoniae* , respiratory syncytial viruses (RSV), human parechovirus, enterovirus, and adenovirus; sub‐typing for RSV was not conducted.

### 2.4 Descriptive Analysis

Descriptive statistics were used to summarize the demographic, clinical, and virologic characteristics of tested ILI and SARI cases, including disaggregation by age. Continuous variables were summarized using median and interquartile range (IQR), while categorical variables were summarized using numbers and percentages. Statistical comparisons were made with a Bonferroni‐corrected *p*‐value using a chi‐squared test or a Fisher's exact test when cell counts were < 5, and Monte Carlo simulations were conducted when comparisons had multiple categories. SPSS and R (v.4.4.0) software were used to perform statistical analyses and data visualizations.

### 2.5 Ethical Considerations

Because these data were collected for public health surveillance, this activity was deemed non‐research and additional ethical clearance was not required, per applicable Tunisia laws. This activity was reviewed by the US CDC, deemed not research, and was conducted consistent with applicable federal law and US CDC policy (45 C.F.R. part 46.102(l)(2), 21 C.F.R. part 56; 42 U.S.C. §241(d); 5 U.S.C. §552a; 44 U.S.C. §3501 et seq.).

### 2.6 Funding

There were no external funds for this analysis. Reagents and consumables for surveillance testing were purchased by the Tunisia national Influenza program (Ministry of Health) or provided to the NIC, free of cost, by the US CDC's International Reagent Resource [16].

## 3 Results

### 3.1 Overview

**FIGURE 2:**
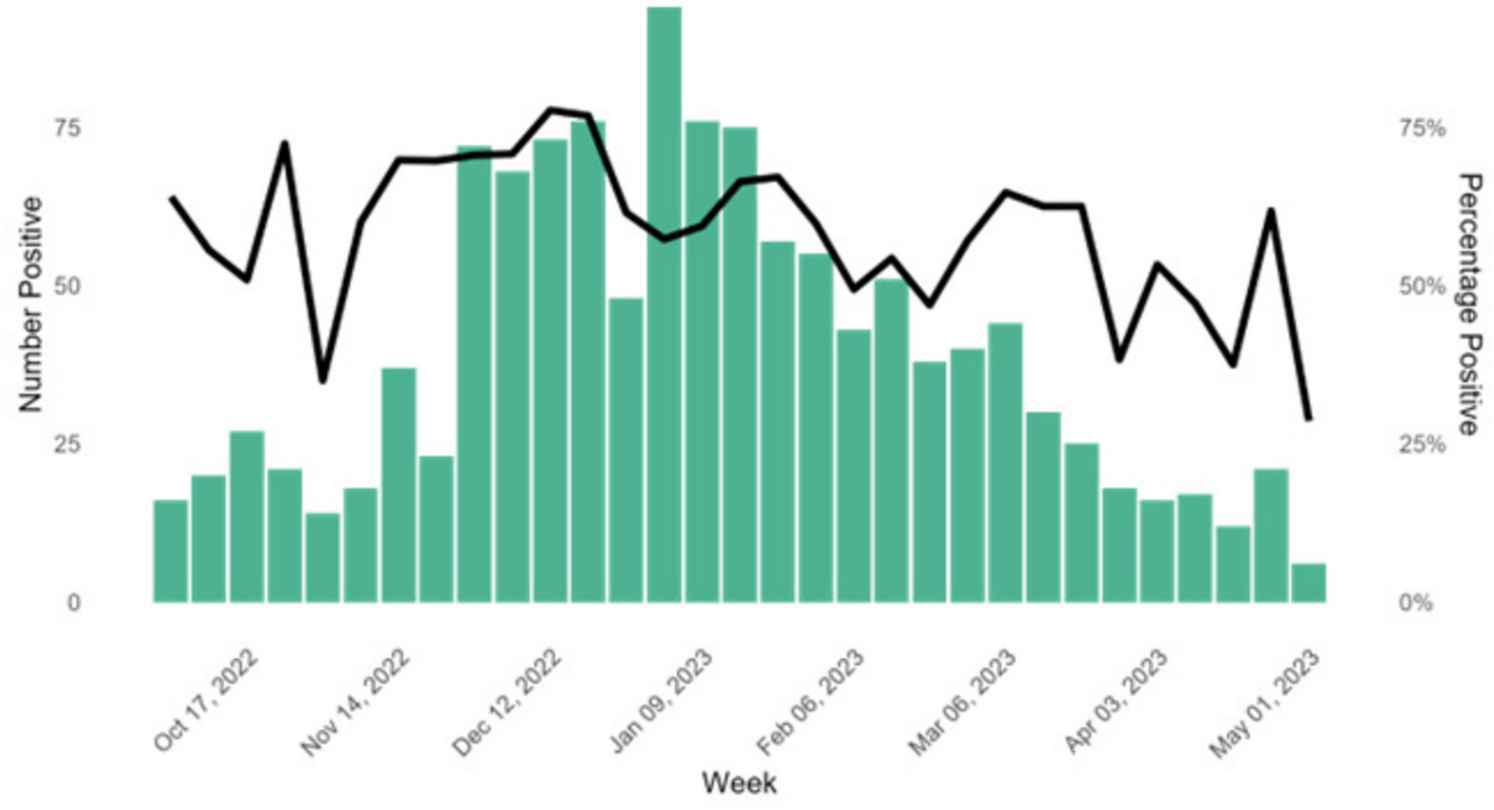
Number and percent of specimens positive for any detected virus, Tunisia, 2022–2023 (*n* = 2038). The bars represent the number of positive specimens, and the line represents the percentage positive. Dates on the x‐axis correspond to 2022 epidemiologic week 40 through 2023 epidemiologic week 20.

During the 2022–2023 season from October to May, the NIC received and tested 2038 specimens from unique case patients from sentinel sites. Of those, 1185 (58.1%) were from SARI cases (Table 1). More than half were from females (*n* = 1079, 52.9%) (sex‐ratio F/M = 1.1). The age of case patients ranged from 1 month to 96 years (median: 43.9 years; IQR: 13.7–64.3 years). The weekly number of specimens received and tested ranged from 21 to 164 (median: 53; IQR: 35–94). The weekly percentage of specimens positive for at least one respiratory pathogen ranged from 28.6 to 77.7% (median: 60%; IQR: 52.1%–66.7%) (Figure 2).

**TABLE 1:** Characteristics of tested influenza‐like‐illness (ILI) and severe acute respiratory infection (SARI) cases and virological results, Tunisia, 2022–2023 (*n* = 2038).

	All tested samples, *N* (%)	ILI (*N* = 853), *n* (%)	SARI (*N* = 1185), *n* (%)	Adjusted *p* *
Sex				
Male	959 (47.1)	292 (34.2)	667 (56.3)	0.02
Female	1079 (52.9)	561 (65.8)	518 (43.7)	
Age group (years)				
< 2	317 (15.6)	26 (3.1)	291 (24.5)	0.02
2–4	59 (2.9)	50 (5.9)	9 (0.8)	
5–14	136 (6.7)	104 (12.2)	32 (2.7)	
15–49	582 (28.7)	363 (42.5)	219 (18.5)	
50–64	382 (18.7)	163 (19.1)	219 (18.5)	
≥ 65	466 (22.9)	92 (10.8)	374 (31.6)	
Missing	96 (4.7)	55 (6.4)	41 (3.4)	
Received influenza vaccine (last 6 months)				
Yes	100 (4.9)	69 (8.1)	31 (2.6)	0.02
No	1938 (95.1)	784 (91.9)	1154 (97.4)	
Received influenza antiviral treatment (last 14 days)				
Yes	30 (1.5)	21 (2.5)	9 (0.8)	0.04
No	2008 (98.5)	832 (97.5)	1176 (99.2)	
Comorbidity				
Cardiovascular diseases	283 (13.9)	75 (8.8)	208 (17.6)	0.02
Asthma	97 (4.8)	31 (3.6)	66 (5.6)	0.86
Other Respiratory diseases	345 (16.9)	25 (2.9)	320 (27.0)	0.02
Diabetes	261 (12.8)	79 (9.3)	182 (15.4)	0.02
Positive virological results				
Positive for at least 1 virus	1231 (60.4)	604 (70.8)	627 (52.9)	0.02
Influenza	445 (21.8)	286 (33.5)	159 (13.4)	0.02
SARS‐CoV‐2	125 (6.1)	53 (6.2)	72 (6.1)	1
Respiratory syncytial virus (RSV)	255 (12.5)	50 (5.9)	205 (17.3)	0.02
Rhinovirus	301 (14.8)	143 (16.8)	158 (13.3)	0.62
Coronaviruses	117 (5.7)	63 (7.4)	54 (4.6)	0.14
Adenovirus	76 (3.7)	36 (4.2)	40 (3.4)	1
Metapneumovirus	64 (3.1)	28 (3.3)	36 (3.0)	1
Parainfluenza	50 (2.5)	26 (3.0)	24 (2.0)	1
Enterovirus	16 (0.8)	7 (0.8)	9 (0.8)	1
Bocavirus	13 (0.6)	3 (0.4)	10 (0.8)	1
Coinfections	200 (16.2**)	76 (12.5**)	124 (19.8**)	0.02

* Bonferroni‐corrected *p*‐values were calculated using the chi‐squared test of independence. A significance level of α = 0.05 was used.

** Percentages of coinfections are calculated among ILI and SARI cases that were positive for at least 1 virus.

In total, 60.4% of specimens (*n* = 1231) were positive for at least one respiratory virus. Influenza was the most detected virus (*n* = 445; 21.8%), followed by rhinovirus (*n* = 301, 14.8%), RSV (*n* = 255, 12.5%), and SARS‐CoV‐2 (*n* = 125, 6.1%) (Table 1). There were no detections of *M. pneumoniae* or parechovirus. We identified 200 (16.2%) specimens that were positive for more than one virus, most of which were positive for two viruses (*n* = 172; 86%) (Tables S1 and S2). There were no discrepancies in influenza virus detections between the US CDC Influenza SARS‐CoV‐2 Multiplex Assay and the FTD‐21 Respiratory Pathogens assay.

### 3.2 Respiratory Virus Circulation

**FIGURE 3:**
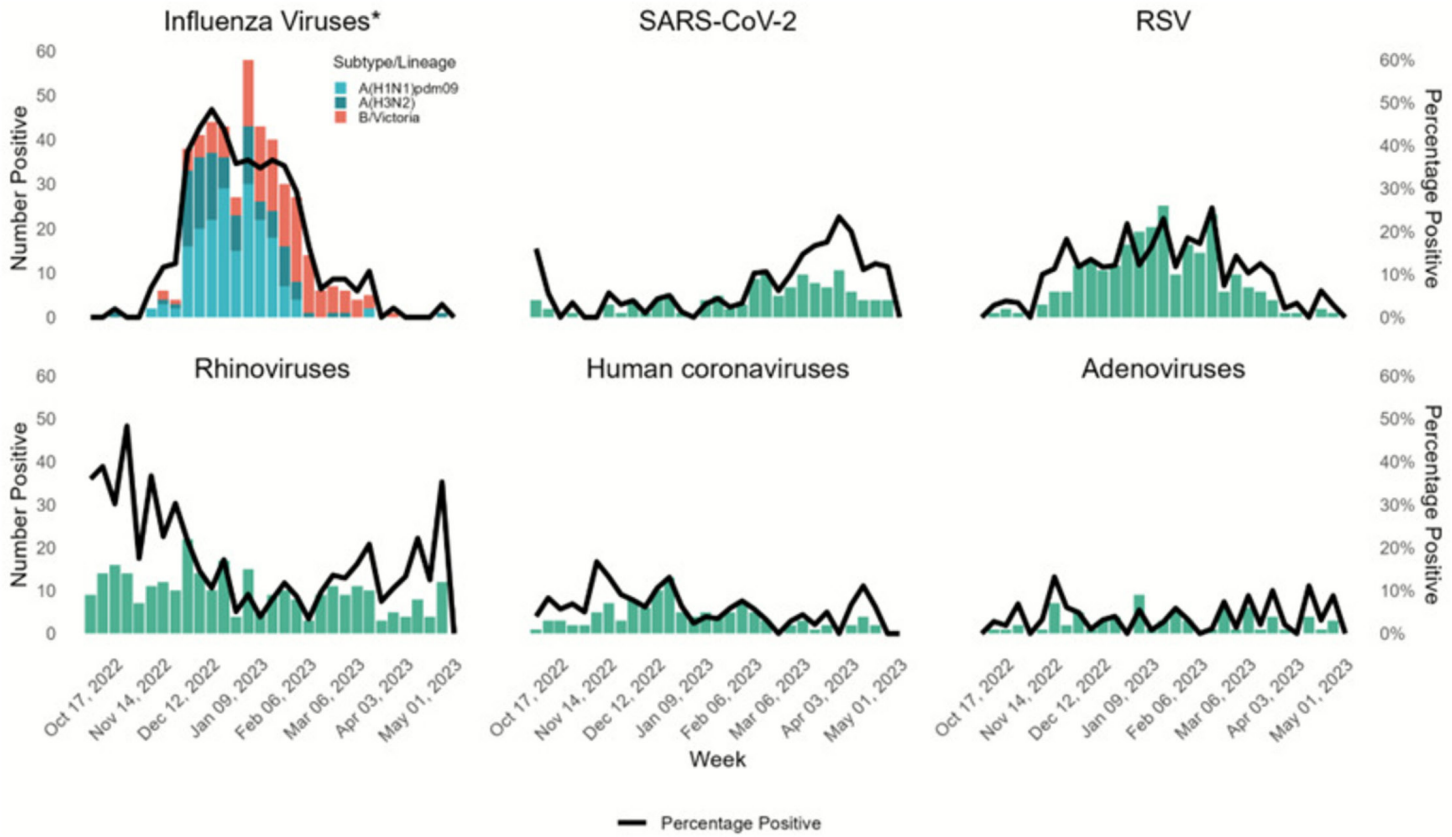
Number and percent of specimens positive for the most detected viral pathogens, 2022–2023, Tunisia. *Three tested specimens were positive for both influenza A and influenza B viruses, shown in the bar graph as separate detections. Weekly percent positivity was calculated as the number of specimens with any influenza detection divided by the total specimens tested. RSV is respiratory syncytial virus. Human coronaviruses exclude SARS‐CoV‐2.

Throughout the season, the weekly percent positivity for influenza viruses ranged from 0–46.8%, with the majority of influenza viruses detected (*n* = 391, 87.9%) occurring between the weeks 48/2022 and 5/2023 (Figure 3 and Figure S1). The highest weekly percentage of influenza‐positive specimens (46.8%) occurred in the week 50/2022. Influenza A viruses predominated earlier in the influenza season (week 42/2022 to week 4/2023), while influenza B viruses predominated later in the season (week 5/2023 to week 13/2023). Of the 445 influenza‐positive specimens, 295 (66.3%) were positive for influenza A viruses only, 147 (33.0%) for influenza B viruses only, and three (0.7%) were coinfected with influenza A and B viruses. Among influenza A positive detections (*n* = 298), 192 (64.4%) were influenza A(H1N1)pdm09, and 106 (35.6%) were influenza A(H3N2). All influenza B detections were Victoria‐lineage.

The weekly percent of specimens positive for RSV ranged from 0% to 25.5% throughout the season. Notably, nearly all weeks between weeks 45/2022 and 12/2023 had at least 10% of specimens positive for RSV (accounting for 96.4% of all RSV detections) (Figure 3). The percent positivity for SARS‐CoV‐2 and rhinoviruses peaked in the weeks before and after peak influenza detections, although the number of cases infected with each virus remained less than 25 and was consistent throughout the season. There were no more than 15 cases a week positive for adenovirus or human coronaviruses other than SARS‐CoV‐2 with relatively stable percent positivity across the season (Figure 3).

### 3.3 Epidemiologic Characteristics of ILI and SARI Cases

SARI cases were more likely than ILI cases to be male (56.3% vs. 34.2%, *p* < 0.02), less likely to have received antiviral treatment for influenza in the previous 14 days (0.8% vs. 2.5%, *p* = 0.04), and less likely to have received an influenza vaccine in the previous 6 months (2.6% vs. 8.1%, *p* = 0.02). The age distribution of ILI and SARI cases varied, where ILI cases tended to be 15–49 years of age compared with SARI cases who were younger (< 2 years) and older (≥ 65 years) (*p* = 0.02). SARI cases were more likely to have a comorbid condition than ILI cases (Table 1).

A higher proportion of ILI cases were positive for at least one virus than SARI cases (70.8% vs. 52.9%, respectively, *p* = 0.02) (Table 1). Influenza viruses were more frequently detected in ILI cases, compared with SARI cases (33.5% vs. 13.4%, respectively, *p* = 0.02), while RSV was more frequently detected in SARI cases compared with ILI cases (17.3% vs. 5.9%, respectively, *p* = 0.02). Of SARI cases with any detected virus, nearly 20% were coinfected with two or more viruses, and 12.5% of ILI cases with a detected virus were coinfected (*p* = 0.02).

**FIGURE 4:**
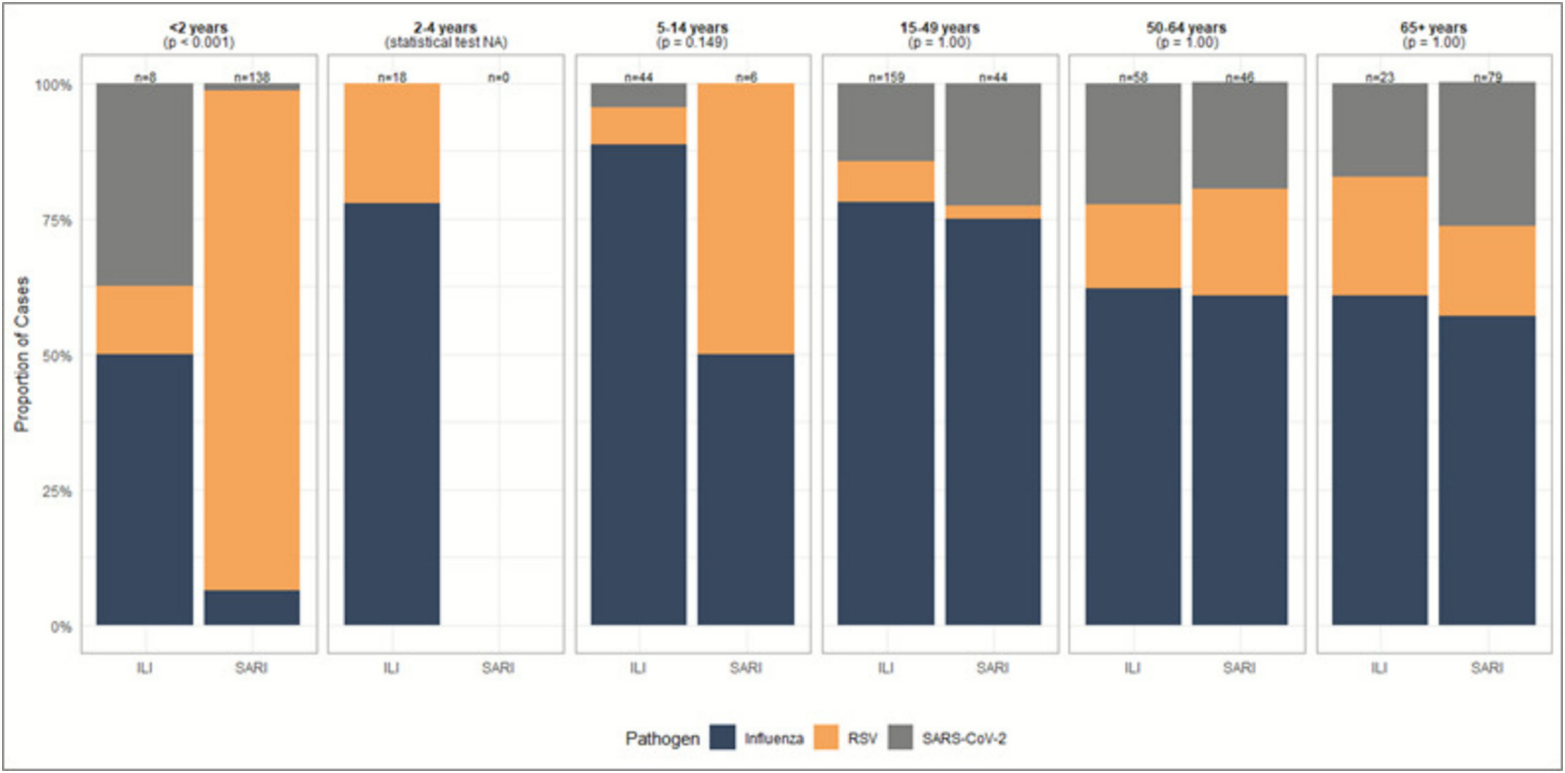
Number and proportion of influenza‐like‐illness (ILI) and severe acute respiratory infection (SARI) cases positive for influenza, SARS‐CoV‐2, or respiratory syncytial virus (RSV) by age group, 2022–2023 Tunisia (ILI *n* = 310, SARI *n* = 313). This figure excludes ILI and SARI cases positive for more than 1 virus, except those with coinfections of influenza A and B. Statistical analysis was done with Bonferroni‐corrected *p*‐values using a chi‐squared test (cell count > 5) or Fisher's exact test (cell count < 5) and a significance level of α = 0.05. An additional 28 ILI cases and 12 SARI cases infected with influenza, SARS‐CoV‐2, or RSV were excluded due to missing age data.

There were differences in case demographics among ILI and SARI cases infected with three key viruses (influenza, SARS‐CoV‐2, and RSV) (Table 2). ILI cases with SARS‐CoV‐2 infections were generally older (93% ≥ 15 years of age) than those with influenza and RSV infections. A higher proportion of ILI cases infected with SARS‐CoV‐2 had at least one comorbid condition compared to ILI cases infected with influenza or RSV. Among SARI cases (*n* = 325), 83.0% of patients with RSV infections were < 2 years of age compared to 7.6% for influenza and 4.8% for SARS‐CoV‐2 infections. When comparing the distribution of viral infections between SARI and ILI cases among the six different age groups, the < 2 years of age showed a strong difference in the distribution with much more influenza among ILI cases and much more RSV among SARI cases (*p* < 0.001) (Figure 4). No other age group had a statistically significant difference in the distribution among RSV, SARS‐CoV‐2, and influenza viruses.

**TABLE 2:** Characteristics of influenza‐like‐illness (ILI) and severe acute respiratory infection (SARI) cases positive for influenza, SARS‐CoV‐2, or respiratory syncytial virus (RSV), 2022–2023, Tunisia^a^.

	ILI									SARI						
	(*n* = 338)									(*n* = 325)						
	All	Influenza		SARS‐CoV‐2		RSV		Adjusted *p*‐value^c^	All	Influenza		SARS‐CoV‐2		RSV		Adjusted *p*‐value^c^
	(*n* = 334)	(*n* = 247)^b^		(*n* = 48)		(*n* = 39)			(*n* = 325)	(*n* = 129)^b^		(*n* = 42)		(*n* = 154)		
		*n*	%	*n*	%	*n*	%			*n*	%	*n*	%	*n*	%	
**Sex**								1								1
Female	228	166	72.8%	35	15.4%	27	11.8%		161	68	42.2%	18	11.2%	75	46.6%	
Male	106	81	76.4%	13	12.3%	12	11.3%		164	61	37.2%	24	14.6%	79	48.2%	
**Age group** (years)								0.04^d^								0.004^d^
< 2	8	4	50.0%	3	37.5%	1	12.5%		138	9	6.5%	2	1.4%	127	92.0%	
2–4	18	14	77.8%	0	0.0%	4	22.2%		0	0	0.0%	0	0.0%	0	0.0%	
5–14	44	39	88.6%	2	4.5%	3	6.8%		6	3	50.0%	0	0.0%	3	50.0%	
15–49	159	124	78.0%	23	14.5%	12	7.5%		44	33	75.0%	10	22.7%	1	2.3%	
50–64	58	36	62.1%	13	22.4%	9	15.5%		46	28	60.9%	9	19.6%	9	19.6%	
65+	23	14	60.9%	4	17.4%	5	21.7%		79	45	57.0%	21	26.6%	13	16.5%	
Missing	28	16	57.1%	5	17.9%	7	25.0%		12	11	91.7%	0	0.0%	1	8.3%	
**Received influenza vaccine** (last 6 months)	22	9	40.9%	9	40.9%	4	18.2%	0.004^d^	11	3	27.3%	4	36.4%	4	36.4%	0.24 ^d^
**Received influenza antiviral treatment** (last 14 days)	3	3	100.0%	NA	NA	NA	NA	—	3	3	100.0%	NA	NA	NA	NA	—
**Comorbidity** (at least one)	66	40	60.6%	15	22.7%	11	16.7%	0.08	122	69	56.6%	32	26.2%	21	17.2%	0.004

Abbreviation: NA: not applicable.

^a^ Excluding ILI and SARI cases positive for more than 1 virus, except those with coinfections of influenza A and B viruses.

^b^ Influenza cases included cases of coinfection of influenza A and B viruses.

^c^ Bonferroni‐corrected *p*‐values calculated using a chi‐squared test and a significance level of α = 0.05, unless otherwise specified.

^d^ Due to small counts (< 5), the Bonferroni‐corrected *p*‐value was calculated using Fisher's Exact with Monte Carlo simulations and a significance level of α = 0.05.

## 4 Discussion

This is the first analysis to describe the viral circulation of multiple respiratory viruses, in addition to influenza, at a national level in Tunisia through sentinel surveillance. Establishing and maintaining a robust surveillance system for influenza and other respiratory viruses is vital to seasonal epidemic and pandemic preparedness nationally and globally. Influenza viruses are known to circulate in Tunisia from the end of October to the end of April with activity typically increasing in mid‐November, peaking in January–February, and influenza A(H1N1)pdm09 typically predominating seasonally since the 2009 H1N1 influenza pandemic [4, 17, 18, 19]. These data indicate that influenza circulation during the 2022–2023 season was generally consistent with historical, pre‐COVID‐19 pandemic data in terms of the timing of the season and the predominant influenza virus subtype, but peak activity, defined as the highest weekly percent positivity, was observed earlier than anticipated in late November 2022. Numerous countries reported influenza viruses starting to circulate earlier than anticipated in 2022, as compared with pre‐pandemic influenza seasons, but this was not seen in Tunisia, as the start of the season was similar to previous years [18, 20, 21]. Additional analyses and more years of post‐pandemic data are needed to evaluate more discrete changes in influenza seasonality in Tunisia and whether these patterns align with other countries with northern hemisphere influenza virus circulation patterns.

We observed differences in the peak activity for different viruses such that rhinovirus activity peaked during weeks with relatively low influenza activity, while RSV and SARS‐CoV‐2 had higher activity later than influenza. Non‐influenza respiratory virus surveillance has not been previously conducted nationwide in Tunisia, and thus, seasonality for other viruses like SARS‐CoV‐2 and RSV has not been established. Ongoing sentinel surveillance of priority respiratory viruses (e.g., influenza, SARS‐CoV‐2, and RSV), including the potential of year‐round monitoring, is important for assessing seasonality and informing vaccination strategies. For example, a limited vaccination program exists in Tunisia for influenza and SARS‐CoV‐2, and surveillance may help better inform priority populations and optimal timing of vaccine administration [22]. Understanding seasonality may also help healthcare systems prepare for the confluence of multiple respiratory viruses by ensuring adequate staffing and supplies.

We detected coinfections of two or more viruses in 16.2% of surveillance specimens. This is higher than reported in Tunisia during 2020–2021 (3.4%), when overall respiratory virus circulation was lower [9]. With the increase of multi‐pathogen testing, coinfections are more likely to be detected. We observed more co‐infections among SARI cases compared with ILI cases, which might suggest increased severity among cases with co‐infections. However, while coinfections could influence viral replication, transmission dynamics, and disease severity [23, 24, 25] (which may burden healthcare systems), many questions remain about whether and which coinfections have clinical significance or lead to increased risk for morbidity and mortality [24, 25].

We found that in the 2022–2023 season, influenza virus infections were detected in more than 20% of patients with acute respiratory illness, and only 5% were recently vaccinated against influenza. Receipt of a recent influenza vaccine was higher among outpatients than those hospitalized (8.1% vs. 2.6%, respectively). Although an influenza vaccine program exists in Tunisia, vaccine coverage and willingness to be vaccinated are low nationally, especially among groups prioritized for vaccination like healthcare workers, pregnant women, and people over 65 years [5, 26, 27, 28]. Vaccination is the most effective way to protect against influenza virus infection and influenza‐associated complications, and this underscores the importance of concerted efforts to increase vaccine uptake and expand the currently limited vaccine program in Tunisia [29].

RSV was the most common virus detected among patients hospitalized for acute respiratory infection, with 83.0% of severe infections occurring in children under 2 years—the age group most at risk for RSV‐attributed morbidity and mortality [30, 31, 32]. Prior to 2023, the only RSV prevention product was a monoclonal antibody that required monthly injections and was cost‐prohibitive for many LMICs [33, 34]. Novel products have been developed, including a vaccine for women during pregnancy and a long‐acting monoclonal antibody for infants, both of which have demonstrated effectiveness, particularly against severe disease, and have the potential to transform the global burden of RSV and reduce morbidity and mortality [34, 35, 36, 37, 38]. Building on robust sentinel surveillance systems like the one described here, additional studies are needed to assess the burden of RSV‐associated hospitalizations and estimate the potential cost–benefit of the introduction of RSV‐prevention products, particularly in LMICs like Tunisia, to guide countries in the development of country‐specific guidelines and policies. These data also highlight the value of global vaccine programs like Gavi, the Vaccine Alliance to promote global distribution of vaccine products so that countries with the highest burden of RSV, which are primarily LMICs like Tunisia, can have access to these products [39].

### 4.1 Strengths and Limitations

This analysis leveraged national sentinel surveillance data in Tunisia across 96 sites, enrolling over 2000 ILI and SARI patients for the 2022–2023 season. The multiplex testing kit allowed testing for 21 respiratory pathogens to provide a comprehensive view of respiratory virus activity after the COVID‐19 pandemic. However, there are some important limitations. Surveillance specimens were only collected during the expected influenza season (October to April) so the circulation of SARS‐CoV‐2, RSV, and other viruses may not be fully captured. Although the surveillance system is nationwide in Tunisia, it is inherently based within public healthcare facilities and may not represent all sub‐populations in Tunisia; in addition, hospitals that serve pediatric populations were overrepresented among SARI hospitals. While all SARI patients were sampled, only a proportion of patients with ILI were sampled and the number sampled varied by site and over time. Because there is no record of patients with ILI who were not swabbed, we are unable to assess whether there was systematic bias of sampling among ILI cases. Thus, ILI cases included in this analysis may not be representative of all people with influenza‐like illness in Tunisia, and the sample size for some age groups was small (e.g., 0–2 years), which limits interpretation for virus prevalence. While active data quality and assurance processes helped prevent missing data, some variables (e.g., pregnancy status, travel history, date of illness onset) were excluded from this analysis because they had more than 30% of missing data. This limited our ability to examine certain relationships; for example, we were not able to look at other factors affecting viral detection such as time from illness onset to sample collection.

## 5 Conclusion and Recommendations

While the public health benefits of routinely testing for a broad spectrum of respiratory pathogens remain unclear, our study highlights the importance of maintaining strong sentinel surveillance of priority respiratory viruses like influenza, SARS‐CoV‐2 and RSV, where interventions such as vaccination can reduce morbidity and mortality. Understanding the circulation patterns of priority respiratory viruses is crucial for comprehensive preparedness and response, nationally and globally, helping to mitigate pressures on the healthcare system during periods of high viral co‐circulation. Increasing influenza vaccine coverage among groups at higher risk for severe disease outcomes should be prioritized, and consideration should also be given to introducing the RSV vaccine to prevent RSV‐associated burden among children.

## Author Contributions

**Bouslah Zoubeir:** writing – original draft, conceptualization, data curation. **Bouguerra Hind:** conceptualization, methodology, formal analysis, writing – original draft, data curation. **Dhaouadi Sonia:** methodology, formal analysis, writing – original draft. **Abid Salma:** data curation, methodology, formal analysis, writing – review and editing, conceptualization. **Whitehouse Erin Rachel:** methodology, writing – review and editing, visualization. **El Ghord Hakim:** data curation, resources. **Yazidi Rihab:** data curation, resources. **Ben Mrad Nacef:** investigation. **Miraoui Amal:** investigation. **Bougharriou Ichrak:** investigation. **Ennouri Emna:** investigation. **Ben Slimen Najla:** investigation. **Guebsi Nour El Houda:** investigation. **Toumi Radhouane:** investigation. **Bouabid Leila:** data curation. **Ben Hamida Chokri:** investigation. **Merhabane Takoua:** investigation. **Boussarsar Mohamed:** investigation. **Ben Jemaa Mounir:** investigation. **Menif Khaled:** investigation. **Ben Khelil Jalila:** investigation. **Ben Alaya Nissaf:** investigation. **Ben Salah Afif:** validation. **Laouini Dhafer:** validation, resources. **Zureick Kinda:** methodology, formal analysis, visualization, writing – review and editing. **Boutiba‐. Ben Boubaker Ilhem:** conceptualization, supervision, validation, writing – review and editing.

## Conflicts of Interest

The authors declare no conflicts of interest.

## Disclaimer

The findings and conclusions in this report are those of the authors and do not necessarily represent the official position of the Centers for Disease Control and Prevention.

## Supporting information


**Table S1:** Coinfections with two pathogens detected among influenza‐like‐illness (ILI) cases, 2022–2023 season, Tunisia (*n* = 62)*
**Table S2:** Coinfections with two pathogens detected among severe acute respiratory (SARI) cases, 2022–2023 season, Tunisia (*n* = 110)*
**Figure S1:** Number and percent of specimens positive for influenza viruses by age group, Tunisia, 2022–2023. The bars represent the number of influenza positive specimens, and the line represents the percentage positive.
**Figure S2:** Number and percent of specimens positive for SARS‐CoV‐2 by age group, Tunisia, 2022–2023. The bars represent the number of SARS‐CoV‐2 positive specimens, and the line represents the percentage positive.
**Figure S3:** Number and percent of specimens positive for respiratory syncytial virus (RSV) by age group, Tunisia, 2022–2023. The bars represent the number of RSV positive specimens, and the line represents the percentage positive.

## Data Availability

Data available on request due to privacy/ethical restrictions.
