# Supplementary material for: Circulation of Influenza and Other Respiratory Viruses in Tunisia, 2022–2023 Season
Source: Influenza Other Respir Viruses. 2026 May 11;20(5):e70199. doi: 10.1111/irv.70199 (PMC13158699; doi:10.1111/irv.70199)

**Supplemental Table 1: Coinfections with two pathogens detected among influenza-like-illness (ILI) cases, 2022-2023 season, Tunisia (n=62)***

|  |  | **Pathogen 2** | | | | | | | | | |
| --- | --- | --- | --- | --- | --- | --- | --- | --- | --- | --- | --- |
|  |  | **Influenza** | **SARS-CoV-2** | **RSV** | **Rhinovirus** | **Human Coronaviruses** | **Metapneumovirus** | **Parainfluenza viruses** | **Adenovirus** | **Enterovirus** | **Bocavirus** |
| **Pathogen 1** | **Influenza** | 0 | 0 | 2 | 11 | 6 | 1 | 0 | 9 | 1 | 1 |
|  | **SARS-CoV-2** | 0 | -- | 0 | 2 | 0 | 0 | 1 | 0 | 1 | 0 |
|  | **RSV** | 2 | 0 | -- | 5 | 1 | 0 | 0 | 0 | 1 | 0 |
|  | **Rhinovirus** | 11 | 2 | 5 | -- | 4 | 3 | 1 | 3 | 2 | 1 |
|  | **Human coronaviruses** | 6 | 0 | 1 | 4 | -- | 1 | 1 | 2 | 0 | 0 |
|  | **Metapneumovirus** | 1 | 0 | 0 | 3 | 1 | -- | 0 | 1 | 0 | 0 |
|  | **Parainfluenza viruses** | 0 | 1 | 0 | 1 | 1 | 0 | -- | 1 | 0 | 0 |
|  | **Adenovirus** | 9 | 0 |  | 3 | 2 | 1 | 1 | -- | 0 | 0 |
|  | **Enterovirus** | 1 | 1 | 1 | 2 | 0 | 0 | 0 | 0 | -- | 0 |
|  | **Bocavirus** | 1 | 0 | 0 | 1 | 0 | 0 | 0 | 0 | 0 | -- |

* An additional 14 ILI cases in which more than two pathogens were detected are not included in this table.

**Supplemental Table 2: Coinfections with two pathogens detected among severe acute respiratory (SARI) cases, 2022-2023 season, Tunisia (n=110)***

|  |  | **Pathogen 2** | | | | | | | | | |
| --- | --- | --- | --- | --- | --- | --- | --- | --- | --- | --- | --- |
|  |  | **Influenza** | **SARS-CoV-2** | **RSV** | **Rhinovirus** | **Human Coronaviruses** | **Metapneumovirus** | **Parainfluenza viruses** | **Adenovirus** | **Enterovirus** | **Bocavirus** |
| **Pathogen 1** | **Influenza** | 2** | 5 | 5 | 5 | 6 | 0 | 0 | 3 | 0 | 0 |
|  | **SARS-CoV-2** | 5 | -- | 5 | 8 | 3 | 4 | 1 | 2 | 0 | 0 |
|  | **RSV** | 5 | 5 | -- | 18 | 8 | 3 | 0 | 3 | 0 | 1 |
|  | **Rhinovirus** | 5 | 8 | 18 | -- | 1 | 1 | 7 | 7 | 3 | 0 |
|  | **Human coronaviruses** | 6 | 3 | 8 | 1 | -- | 0 | 1 | 2 | 0 | 1 |
|  | **Metapneumovirus** | 0 | 4 | 3 | 1 | 0 | -- | 1 | 0 | 0 | 0 |
|  | **Parainfluenza viruses** | 0 | 1 | 0 | 7 | 1 | 1 | -- | 1 | 0 | 0 |
|  | **Adenovirus** | 3 | 2 | 3 | 7 | 2 | 0 | 1 | -- | 2 | 1 |
|  | **Enterovirus** | 0 | 0 | 0 | 3 | 0 | 0 | 0 | 2 | -- | 0 |
|  | **Bocavirus** | 0 | 0 | 1 | 0 | 1 | 0 | 0 | 1 | 0 | -- |

* An additional 14 SARI cases in which more than two pathogens were detected are not included in this table.

** Co-infection with influenza A and B viruses

**Supplemental Figure 1: Number and percent of specimens positive for influenza viruses by age group, Tunisia, 2022-2023.** The bars represent the number of influenza positive specimens, and the line represents the percentage positive.

**
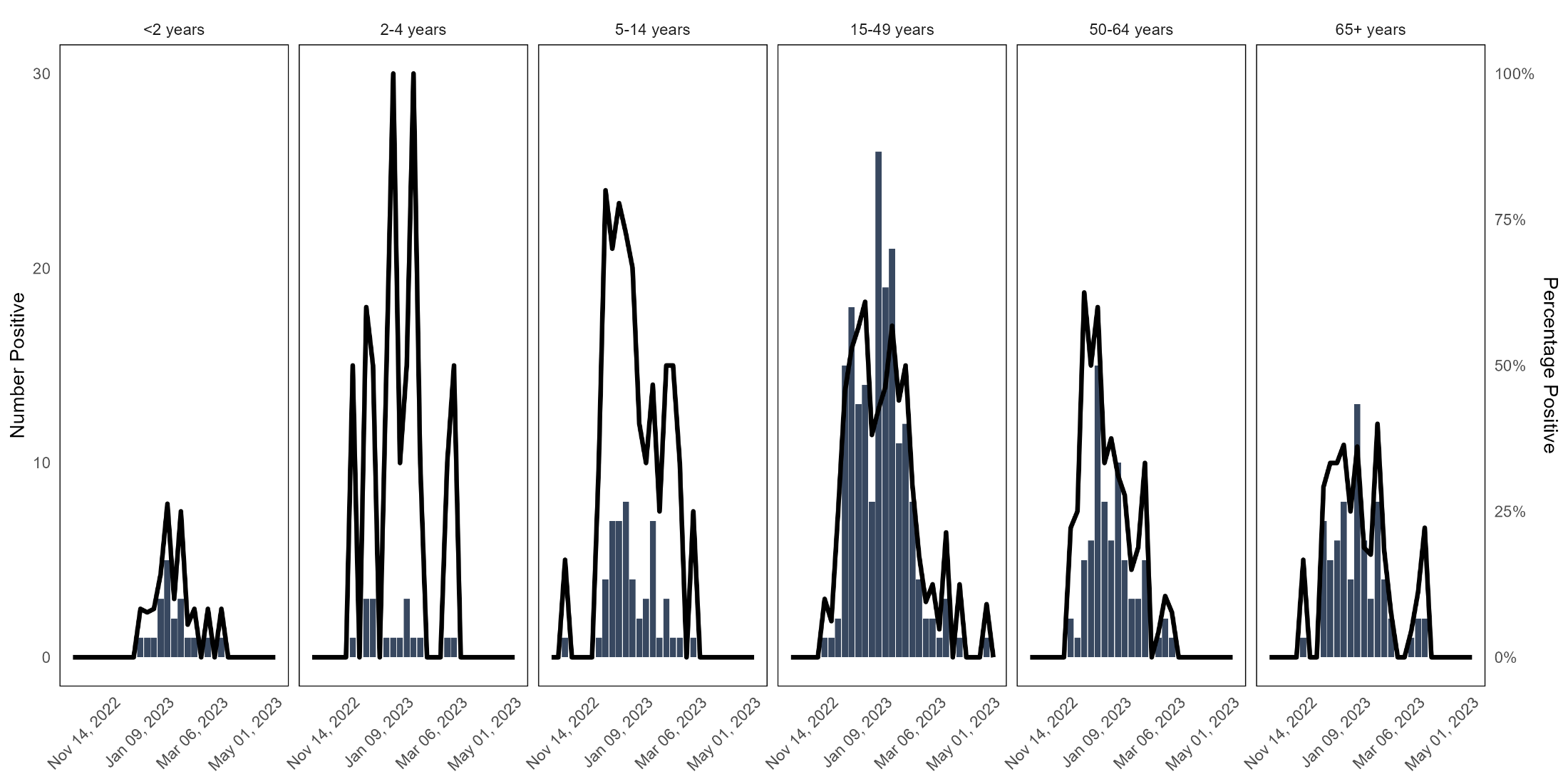
**

**Supplemental Figure 2: Number and percent of specimens positive for SARS-CoV-2 by age group, Tunisia, 2022-2023.** The bars represent the number of SARS-CoV-2 positive specimens, and the line represents the percentage positive.

**
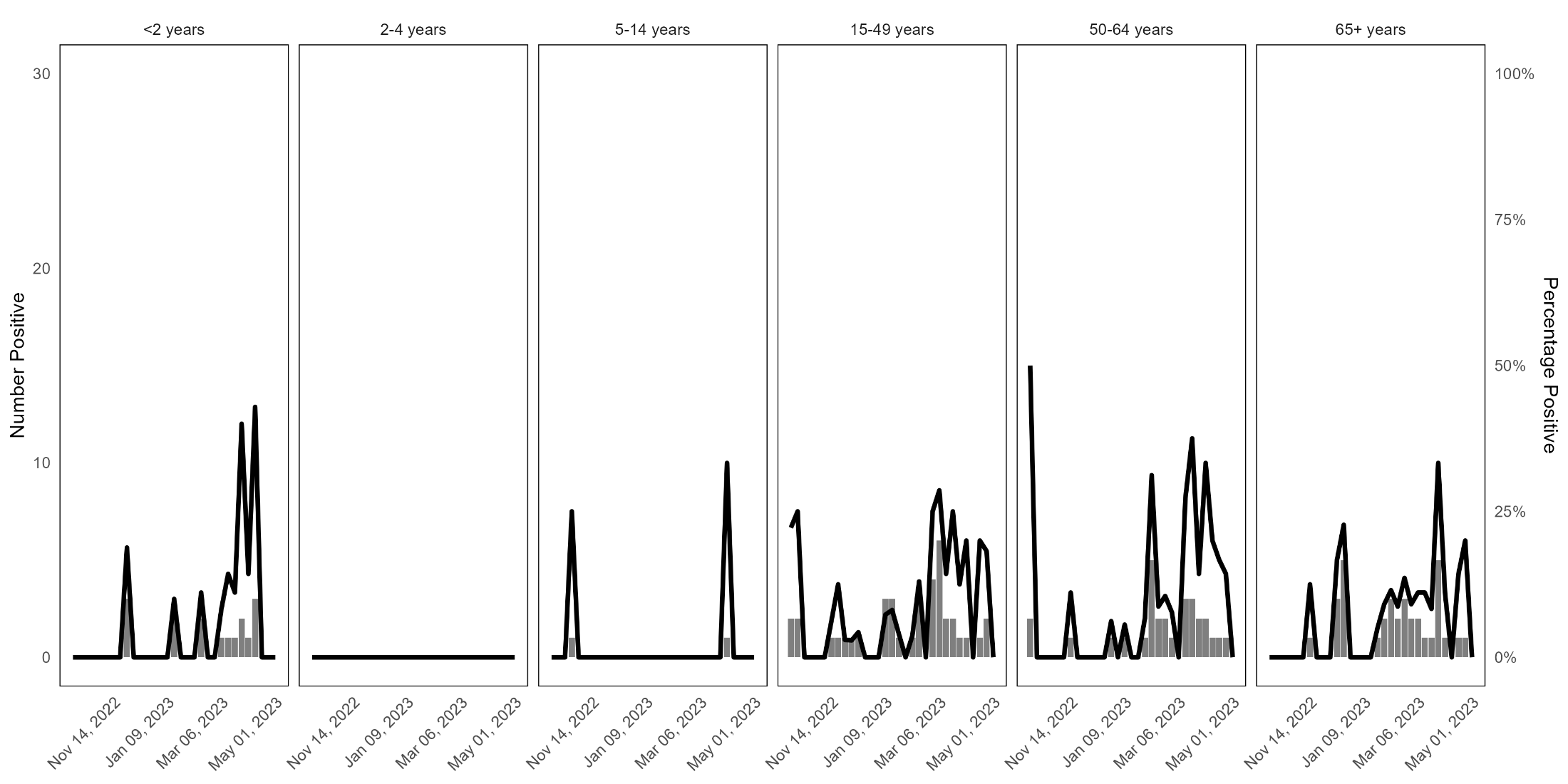
**

**Supplemental Figure 3: Number and percent of specimens positive for respiratory syncytial virus (RSV) by age group, Tunisia, 2022-2023.** The bars represent the number of RSV positive specimens, and the line represents the percentage positive.


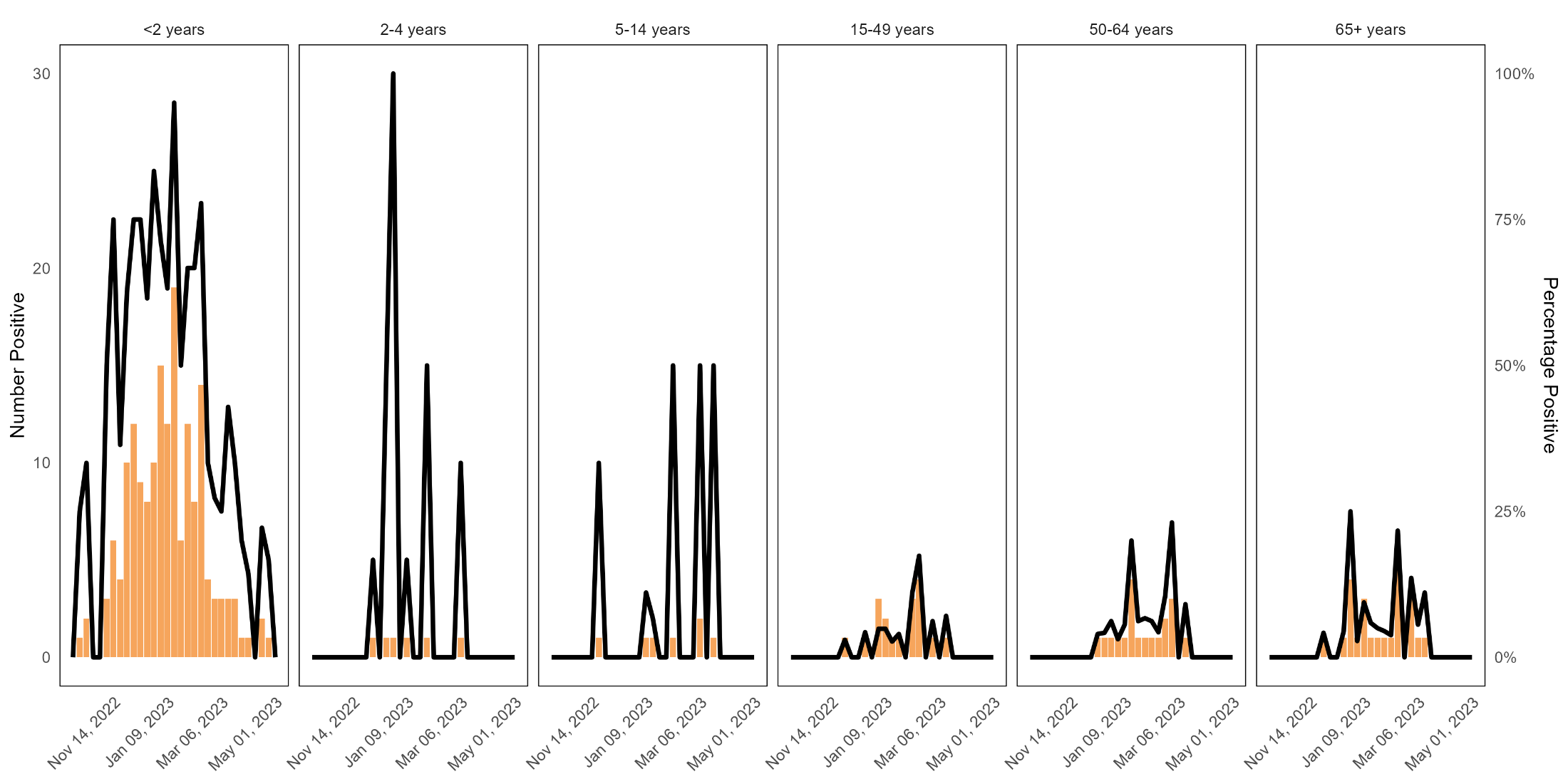

Supplement: Supplementary file 1 — Table S1: Coinfections with two pathogens detected among influenza‐like‐illness (ILI) cases, 2022–2023 season, Tunisia (n = 62)* Table S2: Coinfections with two pathogens detected among severe acute respiratory (SARI) cases, 2022–2023 season, Tunisia (n = 110)* Figure S1: Number and percent of specimens positive for influenza viruses by age group, Tunisia, 2022–2023. The bars represent the number of influenza positive specimens, and the line represents the percentage positive. Figure S2: Number and percent of specimens positive for SARS‐CoV‐2 by age group, Tunisia, 2022–2023. The bars represent the number of SARS‐CoV‐2 positive specimens, and the line represents the percentage positive. Figure S3: Number and percent of specimens positive for respiratory syncytial virus (RSV) by age group, Tunisia, 2022–2023. The bars represent the number of RSV positive specimens, and the line represents the percentage positive. [file IRV-20-e70199-s001.docx]
